# Ceftazidime-Resistant Burkholderia Cepacia: An Unusual Case in a Pregnant Patient

**DOI:** 10.7759/cureus.1812

**Published:** 2017-11-01

**Authors:** Rizwan Ishtiaq, Ali Akram, Laraib Zulfiqar, Daniyal Ishtiaq, Anam Ashraf, Mariam Arif

**Affiliations:** 1 Gastroenterology, Beth Israel Deaconess Medical Center, Boston; 2 Internal Medicine, BIDMC, Harvard Medical School; 3 General Medicine, Quaid-e-Azam Medical College, Bahawalpur, Pakistan; 4 General Medicine, Rawalpindi Medical College, Rawalpindi, Pakistan; 5 Internal Medicine, Rawalpindi Medical College, Rawalpindi, Pakistan; 6 Internal Medicine, DHQ Teaching Hospital Sargodha

**Keywords:** burkholderia cepacia, ceftazidime, drug resistance

## Abstract

Intensive care unit (ICU) sepsis in patients is a common clinical practice primarily in the tertiary care settings. Multidrug resistance to pathogens causing ICU sepsis is widespread, and it poses a severe threat to physicians in terms of managing their patients. At times, physicians get exposed to a pathogen they have never encountered before. Burkholderia cepacia infection in immunocompetent patients is rare. This infection is common in patients with immunocompromised immunity and cystic fibrosis. We report a case of a 34-year-old female who was diagnosed with ceftazidime-resistant Burkholderia cepacia in an ICU setting. This is the first case of drug-resistant Burkholderia cepacia reported from Pakistan.

## Introduction

Burkholderia cepacia is a non-lactose fermenting, catalase-producing, gram-negative bacteria. This bacterium is notorious for causing respiratory tract infections in patients with compromised immunity, cystic fibrosis, or any underlying malignancy [1]. Infection caused by Burkholderia cepacia in immunocompetent patients is extremely rare and very few cases have been reported in the literature. We present a case of a 34-year-old female who was found to have signs of respiratory tract infection followed by Burkholderia sepsis in a healthcare setting. Despite our utmost efforts, we could not save the patient because the pathogen was found to have resistance to ceftazidime. Informed consent was obtained from the patient’s husband to report this case.

## Case presentation

A 34-year-old female who was seven months pregnant presented in the emergency department with complaints of a headache, high blood pressure, and convulsions for one day. She was diagnosed with eclampsia, and treatment with magnesium sulfate and hydralazine was initiated. Her condition worsened on the fourth day of her admission as she became jaundiced. Her fetus was delivered, and a referral to a tertiary care hospital was made.

On examination, the patient was lying unconscious with jaundice, pallor, bruising, and pedal edema. She had a respiratory rate of 24/min, a blood pressure of 90/48 mm Hg, and a pulse of 126/min, which was regular with good volume and no radio-radial or radio-femoral delay. Systemic examination was unremarkable, including the cardiovascular, gastrointestinal, and central nervous systems. Respiratory system examination was positive for left lower lobe crepitation.

Her laboratory investigations revealed a total leukocyte count (TLC) of 26,000 cells per microliter, hemoglobin - 8.7 g/dL, platelets - 67,000 per microliter, creatinine - 5.7 mg/dL, blood urea nitrogen - 141 mg/dL, total bilirubin - 7.2 mg/dL, alanine aminotransferase - 1,655 IU/l, aspartate aminotransferase - 1,295 IU/l, albumin - 2.5 g/dL, international normalized ratio (INR) - 1.6, activated partial thromboplastin time (aPTT) of 56 seconds, and a prothrombin time (PT) of 19 seconds. 

A blood culture was sent, and an intravaginal ultrasound was done, which was negative for any retained product of conception. She was put on piperacillin/tazobactam empirically. Her respiratory distress worsened over the next day; a decision was made to put her on mechanical ventilation because of acute respiratory distress syndrome (ARDS). Chest X-ray showed bilateral infiltration with a PaO2/FiO2 ratio of 237.

Over the coming days, her leukocytosis worsened with a TLC up to 46,000 per microliter, and her condition became critical with deranged renal function tests, liver functions tests, prothrombin time, and international normalized ratio (INR). Her antibiotic was changed to meropenem. On the fifth day, the patient was weaned off from the ventilator, but her sepsis did not improve. On the seventh day, her blood culture came out positive for ceftazidime-resistant Burkholderia cepacia. Though she was getting a sensitive antibiotic, her TLC kept rising with hepatic and renal failure, and she died on the ninth day of her admission with multiorgan failure.

## Discussion

The respiratory tract is the most common route for an infection by Burkholderia cepacia, followed by intravascular catheters [2]. Our patient appeared to have an infection in the lungs accompanied by bacteremia. Burkholderia cepacia can also be spread directly or indirectly from saliva or fomites of patients with cystic fibrosis [3]. Risk of spread is higher by direct exchange of respiratory secretions due to kissing or intimate social contact [4]. Although it is unknown whether our patient might have been exposed to a family member with cystic fibrosis, this might explain the source of the infection to our patient.

In the present case, the initial symptoms of headache, convulsions, and high blood pressure can be explained by eclampsia. After having indwelling catheters, she developed left lower lobe crepitation and bacteremia. She was put on broad-spectrum antibiotics and assisted ventilation. With the help of culture and sensitivity testing, we were able to identify ceftazidime-resistant Burkholderia cepacia, but we were unable to identify the source of infection. We thought of bacteremia due to indwelling catheters. The drug of choice for the empirical treatment of Burkholderia cepacia bacteremia, in this case, was meropenem as it also covered the other suspected causes of sepsis. Despite the use of sensitive antibiotics, her sepsis did not improve and ended in multiorgan failure.

Most of the infections caused by Burkholderia cepacia are found in immunocompromised patients with opportunistic infections and especially those with HIV infection and cystic fibrosis [5]. Antony, et al. reported an outbreak of Burkholderia cepacia bacteremia in a pediatric intensive care unit in South India [6]. The source of this outbreak was found to be contaminated distilled water. Another study from a tertiary care hospital in Riyadh, Saudi Arabia identified an ultrasound probe gel as the source of an outbreak of a Burkholderia infection [7].

Burkholderia cepacia has been found to be resistant to most antibiotics, and treatment should be initiated with a combination of antimicrobials [5]. Antibiotics to which Burkholderia cepacia has been found sensitive to are ceftazidime (95%), cefotaxime, minocycline, and piperacillin. Antibiotics resistant to Burkholderia cepacia are tetracycline, ticarcillin, and aminoglycosides. In our case, it was resistant to ceftazidime.

A study conducted by Tseng, et al. revealed that amongst the 18 isolates of Burkholderia cepacia that they had with resistance to ceftazidime, 17 demonstrated an efflux pump activity [8]. Ribonucleic acid (RNA) sequencing revealed that the overexpression of resistance-nodulation-division (RND)-3 pump activity was attributed to mutations in the efflux pump regulator gene. This can account for the mechanism of ceftazidime resistance in this pathogen. In a recent study, it has been found that avibactam can restore the activity of ceftazidime in ceftazidime-resistant Burkholderia species [9]. A combination of avibactam and ceftazidime was approved in 2015 by the Food and Drug Administration (FDA) for treatment of infections like urinary tract infections caused by multidrug-resistant Enterobacteriaceae. Avibactam is a non-β-lactam β-lactamase inhibitor. The success of this combination is due to the ability of avibactam to inhibit class A and C β-lactamases, including class A carbapenemases (e.g., Klebsiella pneumoniae carbapenemase (KPC)-2) [10]. Ceftazidime-resistant Burkholderia cepacia should be kept in the differentials of the sepsis patient who initially presents with respiratory complaints so that appropriate investigations can be performed in time to improve the treatment outcome.

## Conclusions

The emergence of Burkholderia cepacia sepsis in cystic fibrosis patients, especially in a healthcare setting, poses a significant threat to our community. More and more cases infected with Burkholderia cepacia are being reported, and this pathogen is becoming an increasingly common source of infection in healthcare settings. A high index of suspicion is required to diagnose and treat this pathogen to prevent fatal outcomes related to its disease course.
